# Maternal Morbidity following Trial of Labor after Cesarean in Women Experiencing Antepartum Fetal Death

**DOI:** 10.1007/s43032-024-01645-1

**Published:** 2024-07-11

**Authors:** Ela Kadish, Tzuria Peled, Hen Y. Sela, Ari Weiss, Shaked Shmaya, Sorina Grisaru-Granovsky, Misgav Rottenstreich

**Affiliations:** 1 Department of Obstetrics and Gynecology, Shaare Zedek Medical Center, affiliated with the Hebrew University School of Medicine, Jerusalem, Israel; 2 Faculty of Medicine, Hebrew University of Jerusalem, Jerusalem, Israel; 3 Department of Nursing, Jerusalem College of Technology, Jerusalem, Israel

**Keywords:** Cesarean delivery, Fetal death, Fetal demise, Stillbirth, Trial of labor, Uterine rupture

## Abstract

This study aims to investigate whether trial of labor after cesarean delivery (TOLAC) in women with antepartum fetal death, is associated with an elevated risk of maternal morbidity. A retrospective multicenter. TOLAC of singleton pregnancies following a single low-segment incision were included. Maternal adverse outcomes were compared between women with antepartum fetal death and women with a viable fetus. Controls were matched with cases in a 1:4 ratio based on their previous vaginal births and induction of labor rates. Univariate analysis was followed by multiple logistic regression modeling. During the study period, 181 women experienced antepartum fetal death and were matched with 724 women with viable fetuses. Univariate analysis revealed that women with antepartum fetal death had significantly lower rates of TOLAC failure (4.4% vs. 25.1%, p < 0.01), but similar rates of composite adverse maternal outcomes (6.1% vs. 8.0%, p = 0.38) and uterine rupture (0.6% vs. 0.3%, p = 0.56). Multivariable analyses controlling for confounders showed that an antepartum fetal death vs. live birth isn't associated with the composite adverse maternal outcomes (aOR 0.96, 95% CI 0.21–4.44, p = 0.95). TOLAC in women with antepartum fetal death is not associated with an increased risk of adverse maternal outcomes while showing high rates of successful vaginal birth after cesarean (VBAC).

## Introduction

Cesarean delivery (CD) remains the most frequently performed major surgical procedure for women [1–4]. A trial of labor after cesarean (TOLAC) following a single CD is a viable option for reducing the rate of repeat CD, despite the associated risk of uterine rupture [5–7]. However, careful patient selection is crucial to minimize the risks associated with TOLAC and to increase rates of successful vaginal delivery after CD (VBAC) [8–10].

Antepartum fetal death has an estimated incidence of 0.6% [11]. Studies reviewing complications associated with the delivery of antepartum fetal death have reported higher rates of chorioamnionitis, malpresentation, shoulder dystocia, postpartum hemorrhage (PPH), and retained placenta [12]. Despite the prevalence of antepartum fetal death, there is a lack of data regarding the optimal mode of delivery for women who have experienced antepartum fetal death and have a history of CD. Furthermore, concerns about maternal safety in this situation, particularly the risk of uterine rupture, have been documented [13].

Several factors may contribute to these concerns. The increased incidence of fetal dystocia during labor could raise the risk of uterine rupture in women attempting TOLAC after experiencing antepartum fetal death [14]. Additionally, the absence of a detectable fetal heart rate, a critical indicator for suspecting uterine rupture and recommended during TOLAC in viable pregnancies [15], poses challenges to the timely diagnosis of uterine rupture in women with antepartum fetal death [16].

Therefore, the purpose of this study is to investigate whether TOLAC in women with antepartum fetal death, in comparison to women with a viable fetus, is associated with an increased incidence of maternal morbidity.

## Material and Methods

### Study Design

This research utilized a retrospective multicenter case–control design, leveraging computerized medical records from two university-affiliated obstetrical centers in Israel, covering the period from 2005 to 2021. These centers accounted for approximately 16% of all deliveries in the state, with an average annual volume of 20,000 births during the study period.

### Study Cohort

The study focused on women with singleton pregnancies attempting TOLAC between 24 and 42 weeks of gestation. Eligible participants had a history of a single low-segment cesarean delivery, while those with non-low-segment incisions, uterine body wall extensions, multiple gestations, placental abruption, placenta accrete spectrum, non-vertex presentation, known Mullerian malformation, or out-of-hospital deliveries were excluded. Cases involved women with antepartum fetal death, while controls consisted of women with a viable fetus. Controls were matched with cases in a 1:4 ratio based on their previous vaginal births and induction of labor rates. The matching was based on previous studies findings regarding risk factors for adverse maternal outcomes among women undergoing TOLAC [17]. Importantly, none of the women in our dataset were included more than once.

### Clinical Protocols

Both obstetrical centers adhered to similar departmental protocols consistent with the TOLAC guidelines of the Israeli College of Obstetrics and Gynecology. Patients meeting the criteria were provided with comprehensive information about the risks and benefits of both TOLAC and repeat cesarean delivery. TOLAC deliveries were overseen by board-certified obstetricians who made real-time decisions regarding labor induction, augmentation, operative vaginal deliveries, and emergent cesarean deliveries. Continuous electronic fetal monitoring was mandatory for women with a viable fetus.

TOLAC protocol was similar throughout the study duration.

### Induction of Labor Protocol

For labor induction, various methods were employed based on cervical conditions and physician discretion. These methods included the use of a double-lumen balloon for cervical ripening, amniotomy, or low-dose oxytocin. Oxytocin induction involved administering a solution of oxytocin 10 IU in 1000 ml Ringer's lactate, starting at a rate of 0.5 mU/min. The infusion rate was incrementally increased by 0.5 mU/min every 20 min until achieving 3–4 contractions per 10 min, with a maximum dose of 20 mU/min. Prostaglandins were not utilized as an induction agent during TOLAC. Our routine practice involves early amniotomy. Notably, that there was no variation in the induction protocol between women with or without fetal demise [18, 19].﻿

### Data Collection and Analysis

Relevant information from electronic medical records, including maternal and neonatal records, was coded and reviewed. Patient identification and personal data were anonymized before analysis to ensure confidentiality. The study was approved by the institutional ethics committee (IRB: 0036–23-SZMC), and patient consent was waived due to the retrospective and de-identified nature of the study.

### Outcomes

The primary outcome focused on a composite adverse maternal outcome, comprising uterine rupture or dehiscence, postpartum hemorrhage, blood product transfusion, maternal ICU admission, laparotomy, and hysterectomy. Secondary outcomes included additional maternal morbidity parameters, such as failed TOLAC, retained placenta, shoulder dystocia, severe perineal tear, chorioamnionitis, puerperal fever, relaparotomy, and hysterectomy.

### Definitions

Uterine rupture was defined as a complete uterine scar rupture with a direct connection between the peritoneal space and the uterine cavity. Dehiscence of the uterine scar was defined as an incomplete uterine scar disruption, with the serosa remaining intact and the fetus, placenta, and umbilical cord contained within the uterine cavity [10]. These diagnoses were made by attending physicians during cesarean delivery or postpartum laparotomy.

Chorioamnionitis was defined by the presence of maternal fever (temperature ≥ 37.8 °C or ≥ 38.0 °C) plus two or more of the five following clinical signs: maternal tachycardia (heart rate > 100 beats/min), fetal tachycardia (heart rate > 160 beats/min), uterine tenderness, purulent or foul-smelling amniotic fluid or vaginal discharge, and maternal leukocytosis (white blood cell count > 15,000/mm^3^) [20].

### Statistical Analysis

Descriptive statistics summarized the characteristics of the study population. Categorical variables were compared using the Chi-square test or Fisher's exact test, while continuous variables were analyzed using the unpaired Student's t-test or Mann–Whitney test, as appropriate. Multivariable logistic regression analysis was conducted to adjust for the association between antepartum fetal death and the composite adverse maternal outcome. Results were reported as adjusted odds ratios (aOR) with 95% confidence intervals (CIs). Statistical significance was set at p < 0.05, and all tests were two-sided. SPSS software (version 25, IBM, Armonk, NY) was used for data analysis.

## Results

**Fig. 1:**
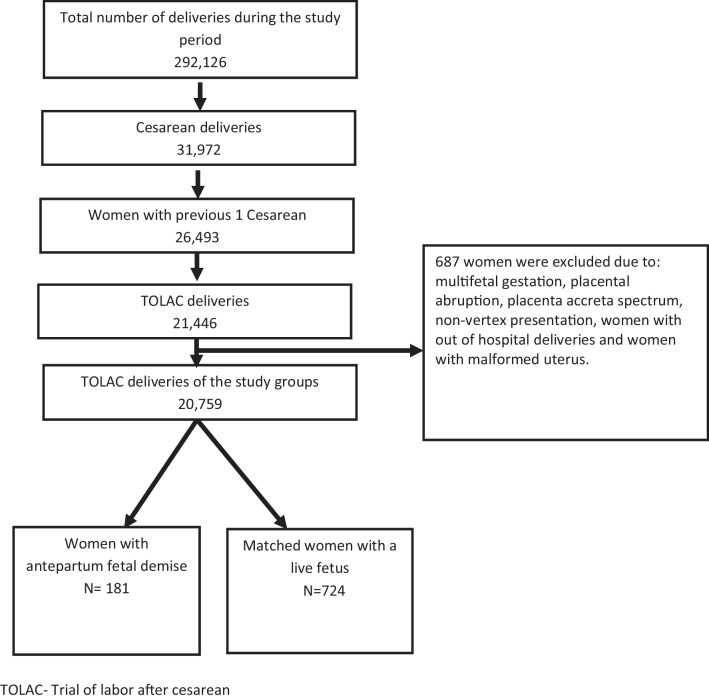
Study Population Schematic Flowchart

A total of 20,759 patients underwent TOLAC and met the inclusion and exclusion criteria during the study period. Among them, 181 (0.6%) experienced a pre-labor fetal death, and these cases were compared to a matched control group of 724 patients with a live fetus (Fig. 1).

Table 1 presents a summary of the general demographic and obstetrical characteristics for both the study and control groups. Women experiencing antepartum fetal death demonstrated higher gravidity and parity, along with lower prevalence rates of diabetes (both pre-gestational and gestational) and disorders of pregnancy. Furthermore, this group exhibited a shorter gestational age at delivery, a higher frequency of balloon cervical ripening, and a reduced incidence of Oxytocin augmentation during labor.

**Table 1:** Demographic and obstetric characteristics of the study and control groups

	Live fetus N = 724	Antepartum fetal death N = 181	P value
Maternal age, years	31.5 ± 5.4	32.4 ± 6.1	0.07
Inter-delivery interval (months)	31.2 ± 16.9	28.3 ± 13.6	0.11
Gravidity	4.9 ± 2.9	5.4 ± 3.3	0.02
Parity	4 ± 2	4.7 ± 2.8	< 0.01
Previous vaginal delivery	532 (73.5%)	133 (73.5%)	1.00
Smoking	21 (3%)	8 (4.9%)	0.24
Fertility treatments	39 (5.4%)	6 (3.3%)	0.25
Hypertensive disorders of pregnancy	29 (4%)	4 (2.2%)	0.25
Diabetes (pre-gestational & gestational)	71 (10.3%)	9 (5.3%)	0.04
Obesity (BMI > 30)	20 (16.3%)	1 (1.9%)	0.01
Anemia, Hb < 11gr/dL on admission	88 (19.3%)	13 (10.8%)	0.03
Induction of labor	404 (55.8%)	101 (55.8%)	1.00
Balloon cervical ripening	44 (6.2%)	43 (24.4%)	< 0.01
Oxytocin augmentation of labor	381 (52.6%)	64 (35.4%)	< 0.01
Epidural analgesia	401 (55.4%)	110 (60.8%)	0.19
Gestational age at delivery (weeks)	34.9 ± 3.8	31.8 ± 6.1	< 0.01
Preterm birth < 37 weeks	349 (48.2%)	122 (67.4%)	< 0.01
Length of first stage of labor, minutes	434.8 ± 394.7	387.7 ± 419.4	0.30
Length of second stage of labor, minutes	31.9 ± 91.8	33.9 ± 58.4	0.80

Data are mean ± standard deviation; number (%). VBAC – Vaginal birth after cesarean

Table 2 illustrates the labor and delivery outcomes for both the study and control groups. Those experiencing antepartum fetal death exhibited a similat rate of composite adverse maternal outcomes (6.1% vs. 8.0%, p = 0.38), with significantly reduced rates of TOLAC failure (4.4% vs. 25.1%, p < 0.01). Notably, the rates for intrapartum cesarean due to failure to progress or other indication that suspected fetal distress was similar between the the study and control groups. There was one instance of maternal ICU admission (attributed to acute fatty liver) and one case of laparotomy (due to uterine rupture diagnosed in the early postpartum period). The rates of uterine rupture were comparable between the groups (0.6% vs. 0.3%, p = 0.56). Furthermore, women with antepartum fetal death experienced statistically shorter hospitalization lengths of stay (2 ± 2.5 vs. 3.7 ± 2.8, p < 0.01).

**Table 2:** Labor and delivery outcomes

		Live fetus N = 724	IUFD N = 181	p-value
Primary outcomes				
Composite adverse maternal outcome*		58 (8%)	11 (6.1%)	0.38
Secondary outcomes				
Uterine rupture		2 (0.3%)	1 (0.6%)	0.56
Dehiscence of uterine scar		0 (0%)	0 (0%)	N/A
TOLAC failure (intrapartum cesarean delivery)		182 (25.1%)	8 (4.4%)	< 0.01
Indication for cesarean delivery	Failure to progress	31 (17%)	2 (25%)	0.69
	Suspected Fetal Distress	57 (31.3%)	0 (0%)	0.06
	Other	105 (57.7%)	6 (75%)	0.33
Chorioamnionitis		25 (3.5%)	7 (3.9%)	0.79
Shoulder dystocia		4 (0.9%)	2 (1.7%)	0.46
Retained placenta/placental fragments		29 (8.5%)	11 (9.6%)	0.72
Perineal tear grade 3/4		2 (0.3%)	0 (0%)	0.48
Puerperal fever		12 (2.6%)	6 (5%)	0.19
Maternal ICU admissions		0 (0%)	1 (0.8%)	0.05
Postpartum hemorrhage		51 (7.4%)	11 (6.5%)	0.67
Blood products transfusion		18 (3.9%)	3 (2.5%)	0.44
Hysterectomy		0 (0%)	0 (0%)	N/A
Laparotomy		2 (0.4%)	1 (0.8%)	0.60
Hospitalization length, days		3.7 ± 2.8	2 ± 2.5	< 0.01
Prolonged hospital admission**		33 (7.2%)	1 (0.8%)	0.01

Data are mean ± standard deviation; number (%)

ICU – intensive care unit; TOLAC – trial of labor following cesarean delivery

^*^ A composite adverse maternal outcome including at least one of the following: postpartum hemorrhage, blood products transfusion, maternal ICU admissions, uterine rupture, dehiscence of the surgical scar, laparotomy, and hysterectomy

^**^ Hospitalization > 7 days after cesarean > 5 days after vaginal delivery

Multivariable logistic regression analysis was conducted to assess the association between antepartum fetal death vs. live birth and composite adverse maternal outcomes. The analysis revealed that antepartum fetal death was not significantly associated with composite adverse maternal outcomes (adjusted odds ratio [aOR] = 0.96, 95% CI 0.21–4.44, p = 0.95) (Table 3).

**Table 3:** Multivariate logistic regression analysis for the association between the antepartum fetal death and composite adverse outcomes *

	p-value	aOR	95%CI	
Intrapartum cesarean delivery	0.01	7.26	1.54	34.32
Parity	0.02	0.57	0.35	0.92
Anemia, Hb < 11gr/dL on admission	0.03	4.35	1.14	16.54
Gravidity	0.07	1.33	0.97	1.80
Obesity (BMI > 30)	0.12	0.15	0.01	1.65
Gestational age at delivery	0.56	1.05	0.89	1.24
Induction of labor	0.93	1.07	0.23	5.06
Antepartum Fetal Death	0.95	0.96	0.21	4.44
Diabetes (Pregestational & gestational)	1.00	N/A	N/A	N/A

CI Confidence Interval, aOR adjusted odds ratio, * A composite adverse maternal outcome including at least one of the following: postpartum hemorrhage, blood products transfusion, maternal ICU admissions, uterine rupture, dehiscence of the surgical scar, laparotomy, and hysterectomy

## Discussion

### Principal Findings

The management of delivery in cases with an antepartum diagnosis of fetal death is a significant clinical concern, yet limited evidence exists in the medical literature. In this retrospective case–control study, we investigated the maternal morbidity associated with TOLAC in women with antepartum fetal death. Our findings indicate that women with antepartum fetal death who attempted TOLAC did not experience an increased risk of maternal morbidity compared to women with a viable fetus attempting TOLAC. In addition, women with antepartum fetal death demonstrated overall higher rates of TOLAC success and shorter hospital stays. These results suggest that TOLAC can be safely pursued in women with antepartum fetal death, with favorable outcomes in terms of successful vaginal delivery and efficient hospitalization.

## Results

We found similar rates of uterine rupture and dehiscence of the uterine scar in women. The incidence of uterine rupture during TOLAC in women with a live fetus varies according to clinical circumstances and has been reported to range between 0.5% and 1% [21–23]. A previous study reported a uterine rupture rate of 2.4% in women diagnosed with a stillbirth who underwent TOLAC during the third trimester [13]. Other studies assessing the incidence of uterine rupture for TOLAC in cases of antepartum fetal death during the second and third trimesters reported rates of 3.7% to 4.8% [24, 25]. One potential reason for this significant difference may be the use of different induction methods. In the aforementioned studies, the majority of patients were induced using prostaglandin agents. Misoprostol is the most commonly used agent for labor induction in cases of stillbirth and is more effective than oxytocin [13, 24, 26–28]. Misoprostol has also shown high efficacy for induction in cases of previous cesarean delivery, with a 91.1% vaginal birth rate [29] and low rates of uterine rupture when used during the second trimester [30, 31]. However, it should be noted that the use of prostaglandin agents, such as Misoprostol, for labor induction in patients with stillbirth has been associated with a higher risk of uterine rupture, as compared to oxytocin induction during the third trimester [32, 33]. Consequently, the American College of Obstetricians and Gynecologists (ACOG) recommends against the use of Misoprostol in term patients who have undergone a CD or major uterine surgery [15]. Nevertheless, in cases of stillbirth, some clinicians still choose to use Misoprostol [34]. In our cohort, which included third-trimester patients with stillbirth, only mechanical methods or oxytocin were employed for induction of labor. The incidence of uterine rupture in the group of women with stillbirth (0.6%) was comparable to that of women with a live fetus. Therefore, until further studies are conducted comparing the safety and efficacy of Misoprostol versus Oxytocin for IOL in third-trimester patients with stillbirth and prior CD, the use of Misoprostol should be approached with caution.

In the present study, there was a single case of uterine rupture observed among women with antepartum fetal death, which was missed during labor and only identified a few hours later. It is widely recommended to use continuous electronic fetal monitoring (EFM) during TOLAC to monitor for significant fetal heart rate decelerations or bradycardia, which may be the earliest sign of uterine rupture [23, 35]. The lack of EFM use in cases of fetal death may increase the risk of uterine rupture and may lead to a delay in diagnosis [36]. Approximately 20% of rupture cases have been reported to have a delayed diagnosis, which is associated with significantly worse outcomes, including higher rates of hysterectomy [37]. Maintaining a high index of suspicion, particularly in cases involving epidurals, is important when EFM is absent [36].

In our study, we found that parturients with antepartum fetal death had significantly lower rates of intrapartum CD, which refers to TOLAC failure. This finding aligns with previous reports [29] and can be attributed to the fact that non-reassuring fetal heart rate, a primary indication for intrapartum CD in cases with a live fetus, does not apply to antepartum fetal death cases [38, 39] as can be seen by similar rates of intrapartum CD due to other indications.

### Clinical and Research Implications

This study highlights the importance of evidence-based management of deliveries with a prior diagnosis of fetal death. The findings suggest that antepartum fetal death does not increase the risk of adverse maternal outcomes. However, careful monitoring for maternal symptoms and a high index of suspicion should be implemented to detect any potential complications. The choice of induction agents for labor induction in cases of stillbirth is crucial. While misoprostol has shown high efficacy in previous studies, it is associated with a higher risk of uterine rupture. Based on our experience, the IOL using mechanical methods or oxytocin resulted in a low rate of failed TOLAC, along with an acceptable rate of uterine rupture. based on our findings TOLAC should be encouraged in women with prior CD and with antepartum fetal demise.

### Strengths and Limitations

This study possesses several notable strengths. First, it comprised a large population, encompassing over 15% of all national deliveries. The study incorporated real-time data validation, ensuring the accuracy and reliability of the collected data. Furthermore, the inclusion of only in-hospital data without transfers helped mitigate potential selection biases. The implementation of a strict department protocol for TOLAC, consistent level of care, and decision-making during the labor and delivery process, including the controlled use of oxytocin, ensured clinical standardization and quality patient care.

This study has several important limitations that should be acknowledged. Firstly, the study group size may limit the generalizability of the findings. Another significant limitation stems from the case–control design employed in this study. Retrospective studies are inherently limited by their reliance on existing data. Furthermore, the study groups exhibited non-comparable background parameters, which could introduce confounding factors that may impact the outcomes. Although we addressed this issue through multivariate analysis for the primary outcome, residual confounding effects may persist [40]﻿. Additionally, during the matching of cases and controls, our focus was on the presence of a 'previous vaginal delivery' without differentiation between deliveries occurring before or after the CD. We recognize that whether this 'previous vaginal delivery' occurred before or after the CD may influence the rates of TOLAC failure and the risk for uterine rupture﻿ [41]. Unfortunately, this specific information was not available to us, constituting a limitation in our study.

## Conclusions

Our study provides insights into the management and maternal outcomes of deliveries with a prior diagnosis of fetal death attempting TOLAC. Our findings suggest that antepartum fetal death does not appear to increase the risk of adverse maternal outcomes, with high rates of successful vaginal birth. This provides reassurance to clinicians and patients considering TOLAC in the context of fetal death. These findings highlight the importance of individualized care, close monitoring for potential complications, and adherence to established protocols during labor and delivery for these cases. Further research is needed regarding the ultimate induction of labor agents for this population group.

## Data Availability

Not available.
